# Determining the IC_**50**_ Values for Vorozole and Letrozole, on a Series of Human Liver Cytochrome P450s, to Help Determine the Binding Site of Vorozole in the Liver

**DOI:** 10.1155/2015/321820

**Published:** 2015-11-09

**Authors:** Lendelle Raymond, Nikita Rayani, Grace Polson, Kylie Sikorski, Ailin Lian, Melissa A. VanAlstine-Parris

**Affiliations:** Chemistry Department, Adelphi University, Garden City, NY 11530, USA

## Abstract

Vorozole and letrozole are third-generation aromatase (cytochrome P450 19A1) inhibitors. [^11^C]-Vorozole can be used as a radiotracer for aromatase in living animals but when administered by IV, it collects in the liver. Pretreatment with letrozole does not affect the binding of vorozole in the liver. In search of finding the protein responsible for the accumulation of vorozole in the liver, fluorometric high-throughput screening assays were used to test the inhibitory capability of vorozole and letrozole on a series of liver cytochrome P450s (CYP1A1, CYP1A2, CYP2A6, and CYP3A4). It was determined that vorozole is a potent inhibitor of CYP1A1 (IC_50_ = 0.469 *μ*M) and a moderate inhibitor of CYP2A6 and CYP3A4 (IC_50_ = 24.4 and 98.1 *μ*M, resp.). Letrozole is only a moderate inhibitor of CYP1A1 and CYP2A6 (IC_50_ = 69.8 and 106 *μ*M) and a very weak inhibitor of CYP3A4 (<10% inhibition at 1 mM). Since CYP3A4 makes up the majority of the CYP content found in the human liver, and vorozole inhibits it moderately well but letrozole does not, CYP3A4 is a good candidate for the protein that [^11^C]-vorozole is binding to in the liver.

## 1. Introduction

**Figure 1:**
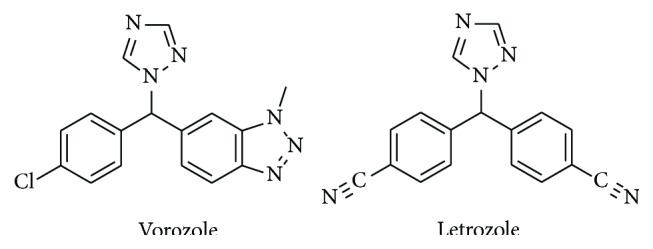
Structures of vorozole and letrozole.

Vorozole and letrozole (Figure 1) are nonsteroidal, triazole-containing compounds that are competitive, reversible, third-generation aromatase (CYP19A1) inhibitors [1, 2]. Both vorozole and letrozole were initially developed and underwent clinical trials as antineoplastic agents [3]. Letrozole (Femara) is currently used in the treatment of breast cancer [4] but vorozole was not pushed forward after phase III clinical trials because it did not show any significant advantage over the current drugs [5–7]. However, vorozole, labeled with ^11^C, is currently being used as a tracer for positron emission topography (PET) imaging to study CYP19A1 distribution in living animals [8–13].

[^11^C]-Vorozole has been shown to display high and specific binding* in vitro* to CYP19A1-rich human placenta [14], human granulosa cells [15], and rat brains [16].* In vivo* studies have shown that [^11^C]-vorozole binds regionally specifically to CYP19A1 in rhesus monkey [16], baboon [11, 17], and human [8] brain. The brain accumulation has been shown to be specific by being blocked by both vorozole and letrozole [8, 16, 17]. However, when [^11^C]-vorozole is administered to rats [10], rhesus monkeys [14], baboons [17], or humans [18] by IV, some of it binds to the liver. It has been shown that this binding in the baboon and human liver is not caused by CYP19A1 because pretreatment with letrozole does not block its binding [18, 19]. While vorozole has been shown to be selective against other cytochrome P450s- (CYP-) dependent reactions in steroid biosynthesis [6], there is limited data available on other CYPs, especially those found in the liver.

Cytochrome P450s are heme containing monooxygenases responsible for oxidative metabolism of more than 95% of pharmaceutical drugs in the human liver. There are a number of xenobiotic metabolizing CYPs that are expressed in a typical human liver and the top CYP isoforms that contribute to the metabolism of small molecule drugs are CYP3A, CYP2C, CYP1A2, CYP2A6, and CYP2E1 [20]. Many imidazole and triazole ring-containing inhibitors of CYPs form a noncovalent ligand interaction with the ferric ion heme and therefore have the potential to inhibit multiple isoforms [21]. Vorozole and letrozole are both triazole-containing compounds so it is likely that they will bind to other CYPs besides CYP19A1.

Since vorozole has been shown to bind to the liver and pretreatment with letrozole does not block this binding, by determining and comparing the binding affinity of both vorozole and letrozole on a series of liver CYPs, we can potentially identify the protein that is responsible for vorozole binding in the liver. This CYP can be identified by having a high binding affinity to vorozole but not letrozole. Fluorometric high-throughput screening (HTS) assays for CYPs have been developed for 13 recombinant human CYPs [22]. These assays use nonnatural coumarin substrates that are converted into fluorescent products by the CYPs. These fluorometric HTS assays can be used to determine the IC_50_ values of vorozole and letrozole on human liver CYPs. By comparing the potency of vorozole and letrozole (as a negative control) on human liver CYPs we can have a better idea of the CYP responsible for vorozole's accumulation in the liver.

## 2. Materials and Methods

### 2.1. Materials

Coumarin, glucose-6-phosphate dehydrogenase, 7-methoxy-4-(trifluoromethyl)coumarin (MFC), 7-hydroxy-4-(trifluoromethyl)coumarin (HFC), magnesium chloride (MgCl_2_), and nicotine adenine dinucleotide phosphate (NADP^+^) were purchased from Sigma Aldrich (St. Louis, MO). 3-Cyano-7-ethoxycoumarin (CEC), 7-benzyloxy-4-(trifluoromethyl)coumarin (BFC), and all recombinant microsomes from baculovirus-infected insect cells (supersomes) were purchased from BD Bioscience (Woburn, MA). 3-Cyano-7-hydroxycoumarin (CHC) was purchased from Indofine Chemical Company. Potassium phosphate dibasic (K_2_HPO_4_) was obtained from Merck. Potassium phosphate monobasic (KH_2_PO_4_), D-glucose 6-phosphate sodium salt, 7-hydroxycoumarin (HC), and dimethyl sulfoxide (DMSO) were purchased from Fisher Scientific Company. Vorozole and letrozole were provided by Brookhaven National Laboratory. All experiments were completed in all black, flat bottom Costar 96-well plates (Corning Incorporated, Corning, NY).

### 2.2. Enzyme Assay

IC_50_ determinations for human CYPs were similar to HTS methods described by Crespi et al. [23]. Unless otherwise stated, the incubations were carried out in a total volume of 200 *μ*L of 50 mM potassium phosphate buffer (pH 7.4) with 1% acetonitrile. The reaction mixtures contained NADPH-regenerating system in potassium phosphate buffer (pH 7.4) and varying amounts of inhibitor (dissolved in 100% acetonitrile) were preincubated at 37°C for 10 minutes (except CYP2A6 which was in 100 mM Tris buffer pH 7.4). The reactions were initiated by the addition of enzyme/substrate mixture (see Table 1), followed by incubation at 37°C for 30 minutes. The fluorescent signal was measured using a SpectraMax GEMINI XPS (Molecular Devices) with excitation and emission wavelengths listed in Table 1. The data were fit to sigmoidal dose-response curves with nonlinear regression and IC_50_ values calculated using GraphPad Prism 5. IC_50_ values were converted to *K*_*i*_ values using the Cheng-Prusoff equation (*K*_*i*_ = IC_50_/(1 + [*S*]/*K*_*m*_)) and literature *K*_*m*_ values when available.

## 3. Results and Discussion

IC_50_ values for vorozole and letrozole on CYP1A1, CYP1A2, CYP2A6, CYP3A4, and CYP19A1 are given in Table 2. Vorozole and letrozole are potent inhibitors of CYP19A1 (IC_50_ = 4.17 and 7.27 nM, resp.). Vorozole is a moderate inhibitor of CYP1A1 (IC_50_ = 0.469 *μ*M) and a weak inhibitor of CYP1A2, CYP2A6, and CYP3A4 (IC_50_ = 321, 24.4, and 98.1 *μ*M, resp.). Letrozole is a weak inhibitor of CYP1A1, CYP1A2, and CYP2A6 (IC_50_ = 69.8, 332, and 106 *μ*M, resp.). Letrozole was an extremely weak inhibitor of CYP3A4 (<10% inhibition at 1 mM).

[^11^C]-Vorozole is currently being used as a radiotracer for CYP19A1 using PET imaging; however when it is administered by IV, some of it binds to the liver but not through CYP19A1. Since vorozole contains a triazole ring and binds to the heme of CYP19A1, it is likely that vorozole can bind to the heme of other CYPs. Since there is a high concentration of CYP protein in the liver, it is likely that vorozole is binding to a CYP other than CYP19A1 in the liver. Letrozole is another potent CYP19A1 inhibitor but it does not block [^11^C]-vorozole's binding in the liver. Therefore we can use letrozole as our negative control when searching for the protein responsible for vorozole accumulation in the liver.

To show that our assays are valid, we confirmed that vorozole and letrozole are very potent inhibitors of CYP19A1. We used the *K*_*m*_ value of 25 *μ*M from the BD Bioscience CYP19A1/MFC kit, to convert our IC_50_ values (4.17 and 7.27 nM) to *K*_*i*_ values of 0.9 and 1.6 nM for vorozole and letrozole, respectively. While there are no literature values for vorozole and letrozole on CYP19A1 with the synthetic substrate MFC, our *K*_*i*_ values are in agreement with the literature *K*_*i*_ values with the natural steroid substrates (testosterone and androstenedione) of around 1 nM for vorozole [24–29] and 2 nM for letrozole [30].

The IC_50_ values for vorozole and letrozole were then compared on each CYP. It was found that vorozole and letrozole bind equally poor to CYP1A2 (1.03-fold difference) with IC_50_ values in the hundred micromolar range. While vorozole is slightly more potent than letrozole on CYP2A6 (4.34-fold difference), they are both still weak inhibitors. Therefore both CYP1A2 and CYP2A6 are not likely candidates for the protein that is causing the accumulation of vorozole in the liver.

On CYP1A1, vorozole was almost 150-fold more potent than letrozole with an IC_50_ of 0.47 *μ*M. However, there is conflicting evidence as to whether CYP1A1 is expressed in the human liver [31]. Even if CYP1A1 is expressed, it is in such small quantities [32] that it is an unlikely candidate.

On CYP3A4, 1 mM letrozole inhibited the reaction by less than 10% so we could not calculate an IC_50_ value. However, vorozole inhibited CYP3A4 with an IC_50_ of 98.1 *μ*M. Therefore, there is at least a 10-fold difference in the potency of vorozole and letrozole on CYP3A4. This data, combined with the fact that CYP3A4 makes up the majority of the CYP content found in the liver [20], implies that CYP3A4 is a good candidate for the enzyme that [^11^C]-vorozole is binding to in the liver but letrozole does not block.

The inhibition potential of vorozole and letrozole on some other liver CYPs has previously been reported in the literature. Letrozole was not a potent inhibitor of CYP1B1 with an IC_50_ ≥ 100 *μ*M for estradiol 4-hydroxylation and 2-hydroxylation. However, vorozole was shown to inhibit CYP1B1 activity with IC_50_ values of 17 and 21 *μ*M for 4-hydroxy estradiol and 2-hydroxy estradiol [33]. Since there is at least a 5-fold difference in potency between vorozole and letrozole and vorozole inhibits CYP1B1 moderately well, CYP1B1 should not be overlooked.

With this information, our collaborators at Brookhaven National Laboratories used a CYP3A4 inhibitor (ketoconazole) to see if it would block the binding of [^11^C]-vorozole to the liver. When [^11^C]-vorozole was given with a pretreatment of ketoconazole, the liver pharmacokinetics of [^11^C]-vorozole binding was altered [19]. While ketoconazole is typically thought of as a selective CYP3A4 inhibitor [34], it has also been shown to inhibit CYP1A1 [35]. This data further confirms that CYP3A4 may be responsible for [^11^C]-vorozole accumulation in the liver but CYP1A1 cannot be completely ruled out.

## 4. Conclusion

In conclusion, this data shows that vorozole is at least 10-fold more potent than letrozole on CYP3A4 and vorozole inhibits CYP3A4 with an IC_50_ value of 98 *μ*M. Therefore CYP3A4 is a likely candidate for the protein responsible for binding vorozole in the liver. Pretreatment with ketoconazole, a CYP3A4 inhibitor, affects liver pharmacokinetics of [^11^C]-vorozole binding, further confirming that CYP3A4 may be responsible for [^11^C]-vorozole accumulation in the liver.

## Figures and Tables

**Table 1:** Fluorometric enzyme assay conditions for recombinant cytochrome P450s (CYPs). 3-Cyano-7-ethoxycoumarin (CEC), 3-cyano-7-hydroxycoumarin (CHC), 7-hydroxycoumarin (HC), 7-benzyloxy-4-(trifluoromethyl)coumarin (BFC), 7-hydroxy-4-(trifluoromethyl)coumarin (HFC), 7-methoxy-4-(trifluoromethyl)coumarin (MFC), excitation wavelength (*λ*_Ex_), and emission wavelength (*λ*_Em_).

Enzyme	Enzyme amount (pmol)	Substrate	Substrate concentration (*μ*M)	Product	*λ*_Ex_ (nm)	*λ*_Em_ (nm)
CYP1A1	1	CEC	5	CHC	410	450
CYP1A2	0.5	CEC	5	CHC	410	450
CYP2A6	1	Coumarin	3	HC	330	460
CYP3A4	1	BFC	50	HFC	409	530
CYP19A1	1.5	MFC	100	HFC	409	530

**Table 2:** IC_50_ values (±SEM) for vorozole and letrozole on CYP1A1, CYP1A2, CYP2A6, CYP3A4, and CYP19A1 for 3 replicate assays.

	Vorozole (*μ*M)	Letrozole (*μ*M)	Fold difference
CYP1A1	0.469 ± 0.031	69.8 ± 5.3	149
CYP1A2	321 ± 49	332 ± 195	1.03
CYP2A6	24.4 ± 3.6	106 ± 34	4.34
CYP3A4	98.1 ± 15.6	<10% @ 1 mM	>10
CYP19A1	0.00417 ± 0.00019	0.00727 ± 0.00070	1.73
